# Tissue Inhibitor of Metalloproteinases-4. The road less traveled

**DOI:** 10.1186/1476-4598-7-85

**Published:** 2008-11-21

**Authors:** Jorge Melendez-Zajgla, Luis Del Pozo, Gisela Ceballos, Vilma Maldonado

**Affiliations:** 1 Instituto Nacional de Medicina Genomica, Mexico; 2 Molecular Biology Laboratory, Instituto Nacional deCancerologia, Av. San Fernando, 22 Tlalpan 14080, Mexico

## Abstract

Tissue inhibitors of metalloproteinases (TIMPs) regulate diverse processes, including extracellular matrix (ECM) remodeling, and growth factors and their receptors' activities through the inhibition of matrix metalloproteinases (MMPs). Recent evidence has shown that this family of four members (TIMP-1 to TIMP-4) can also control other important processes, such as proliferation and apoptosis, by a mechanism independent of their MMP inhibitory actions. Of these inhibitors, the most recently identified and least studied is TIMP-4. Initially cloned in human and, later, in mouse, TIMP-4 expression is restricted to heart, kidney, pancreas, colon, testes, brain and adipose tissue. This restricted expression suggests specific and different physiological functions. The present review summarizes the information available for this protein and also provides a putative structural model in order to propose potential relevant directions toward solving its function and role in diseases such as cancer.

## Background

The extracellular matrix (ECM) not only maintains the three-dimensional structure of tissues and organs, but also plays critical roles in cell proliferation, differentiation, survival and motility. Key to ECM remodeling are the Matrix Metalloproteases (MMPs) and their inhibitors, the Tissue Inhibitors of Metalloproteinases (TIMPs) [1]. These proteins constitute a proteolytic system that participates not only in the breakdown of ECM components and subsequent tissue remodeling, but also provides an important regulatory role in the microenvironment [2]. This is accomplished by the ability of MMPs to modulate the availability and activity of growth factors and cytokines or their receptors, and to process adhesion and signaling receptor targets [3]. For these reasons, it is not unexpected that dysregulation of components of this system are common in diverse pathologies. In particular, it has been found that overproduction of MMPs is associated with cancer initiation and progression in diverse tissues [4]. Although there are several mechanisms that regulate MMP expression, the ultimate control is achieved through interaction with the TIMPs. Intuitively, due to their inhibitory actions, members of this family should be able to inhibit cancer invasion and thus, be antitumoral proteins. Nevertheless, under some circumstances, specific MMPs have a dual role in cancer, with antitumoral activities [1]. In addition, recent evidence has shown that members of the TIMP family can also control other important processes such as proliferation and apoptosis by mechanism(s) independent of their MMP inhibitory actions [5]. This family is composed of four members with high sequence homology and structural identity, but with different tissue expression, regulation and inhibitory characteristics. The most recently identified and least studied member of this family is TIMP-4. Initially cloned in human and later in mouse, TIMP-4 expression is restricted to heart, kidney, pancreas, colon, testes, brain and adipose tissue. This restricted expression suggests specific and different physiological functions.

### Structure

**Figure 1:**
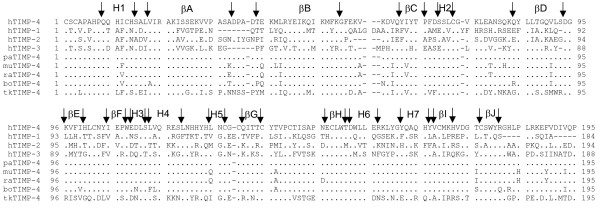
**Comparison of the amino acid sequence of the human TIMP-4 (hTIMP-4) with those of the other human TIMPs (hTIMP-1, hTIMP-2, and hTIMP3) and TIMP-4 from Pan troglodytes (paTIMP-4) Mus musculus (muTIMP-4), Rattus norvegicus (raTIMP-4), Bos taurus (boTIMP-4), and Takifugus rubripes (tkTIMP-4)**. The residues that differ from those encoded by hTIMP-4 are indicated. The secondary structure elements are aligned with the amino acid sequence of the TIMPs and labeled (*H *and β mean "helix" and "β-strand", respectively). The following amino acid sequences were used in the alignment (The GenInfo Identifier numbers, gi, are shown in parentheses): hTIMP-1 (49456917), hTIMP-2 (4507511), hTIMP-3 (47678717), hTIMP-4 (3493223), paTIMP-4 (55619817), muTIMP-4 (110625888), raTIMP-4 (160370007), boTIMP-4 (109886638), tkTIMP-4 (29611415). The alignment was performed with ClustalX (Thompson, J.D., Gibson, T.J., Plewniak, F., Jeanmougin, F. and Higgins, D.G. 1997. The ClustalX windows interface: flexible strategies for multiple sequence alignment aided by quality analysis tools. *Nucleic Acids Research*, 24:4876–4882).

**Figure 2:**
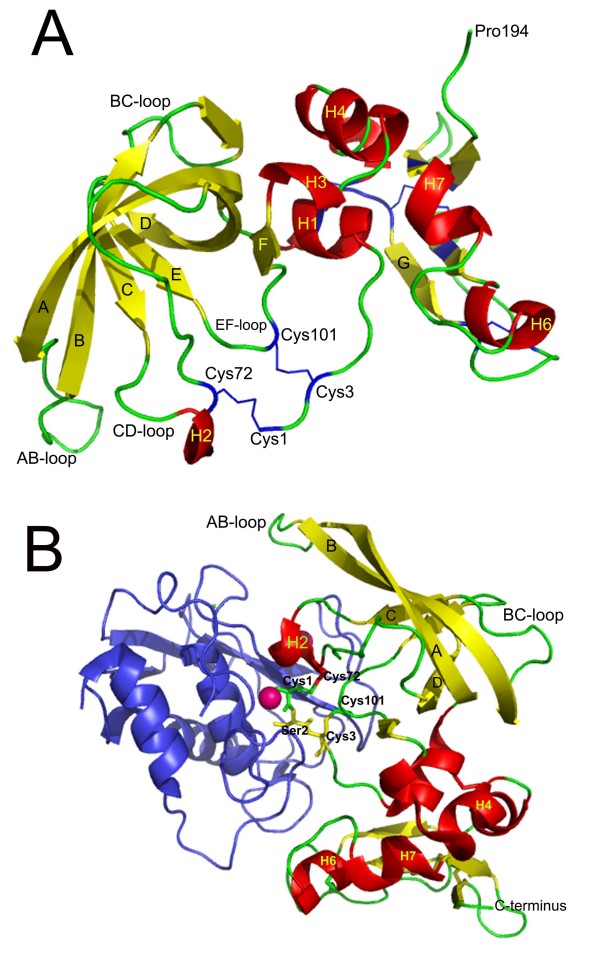
**Cartoon representation of the crystallography structure of the human TIMP-2 in uncomplexed state (A) and the bovine TIMP-2 complexed with the catalytic domain on the metalloproteinase 13 (cdMMP-13) (B)**. The regions of TIMP-2 folded as helices and β-strands are colored in red and yellow, respectively. The six conserved disulfide bridges characterizing the TIMP structure are shown in blue in figure A. In B, the cdMMP-13 is colored in blue and the catalytic Zn is colored in magenta and shown in space-filled format. The residues Cys1, Ser2 and Cys3 that form the core of the molecular edge of the inhibitor that occupies the active-site cleft of the MMP, and the Cys72 and Cys101 are shown in stick format and colored yellow. The figure was prepared using PyMOL^® ^and the coordinates of PDB 1BR9 (Fig. 2A) and PDB 2E2D (Fig. 2B).

Human TIMP-4 is a non-glycosylated, 195 amino acids long polypeptide, the largest of the currently identified human inhibitors of matrix metalloproteinases (MMP) (Fig 1). TIMP-4 and TIMP-2 are 51% identical at the amino acid level, with TIMP-4 only one residue larger than TIMP-2. Contrastingly, TIMP-4 is eleven and seven residues larger than TIMP-1 and TIMP-3 and shows 37% and 51% identity to these proteins, respectively [6-9] (Table 1). Distinctive within the TIMP family, TIMP-4 has twelve Cys residues that form six conserved disulfide bridges [8,10]. The three-dimensional (3D) structure of TIMP-4 has not yet been determined, but due to the relative high sequence identity it shares with the other TIMPs, in particular to TIMP-2, a high structural similarity to those proteins could be expected [7]. The TIMPs fold into two very distinct domains, a larger N-terminal domain that carries the MMP inhibitory activity and a smaller C-terminal domain that mediates other non-inhibitory interactions, notably with some pro-MMP forms (Fig 2A) [8,9,11-13]. The N-terminal domain encompasses nearly two-thirds of the polypeptide chain and is reminiscent of the oligonucleotide/oligosaccharide-binding (OB) fold [14-19]. This motif was first described in proteins binding oligonucleotides or oligossacharides [20] and is present in twelve protein superfamilies within the SCOPE (Structural Classification of Proteins, ) data base, including the staphylococcal nucleases, bacterial enterotoxins, heme chaperone CcmE, N-terminal domain of the tail-associated lysozyme gp5 of bacteriophage T4, nucleic acid-binding proteins and inorganic pyrophosphatases, among others [21,22]. The core of the TIMP OB region is formed by a five-stranded anti-parallel β pleated sheet rolled into a β-barrel, stabilized by three disulfide bonds. The strands forming the barrel are connected by loops, which in some cases differ in length from one TIMP to another. Three segments folded in α-helices (α-helix 1 to 3) are associated with the β-barrel core (Fig 2A) [8,12,14-18,23-26]. The N-terminal domain can be expressed and folded independently and, as it was pointed out above, is necessary and sufficient for MMP inhibition [15,27-29].

**Table 1:** Sequence identity and similarity shown by human and non-human TIMPs compared to the human TIMP-4.

**Protein**	**Identity** **(%)**	**Similarity** **(%)**
hTIMP-1	37	57
hTIMP-2	51	70
hTIMP-3	51	70
paTIMP-4	100	-
muTIMP-4	91	95
raTIMP-4	92	96
boTIMP-4	90	96
tkTIMP-4	55	73
drTIMP*	21	43

The amino acid sequences analyzed are the same as shown in Fig. 1 and the analysis was performed with BioEdit (Hall, T.A., 1999. BioEdit: a user-friendly biological sequence alignment editor and analysis program for Windows 95/98/NT.*Nucl. Acids. Symp. Ser*. 41:95–98).

* Drosophila melanogaster TIMP (gi 4157982)

The C-terminal domain folding topology is characterized by a β-hairpin plus a β-loop-β motif with two associated 3_10_-helices (Fig 2) [14,16,17,26]. Notably, this fold has not been observed in other proteins different from the TIMPs among the structures deposited at the Brookhaven Protein Data Bank ().

Crystallographic structure analysis of the TIMPs in complex with MMPs has shown that the long edge of the wedged-shaped inhibitor occupies the entire length of the MMP active-site cleft. A central part of this molecular edge is formed by a short stretch of five relatively conserved residues at the N-terminus that adopts an extended conformation and a portion of the loop connecting the β-strands C and D (CD-loop) (Fig 2B). The pentapeptide N-terminal segment is connected to the core of the β-barrel by two disulfide bridges involving Cys1-Cys70 and Cys3-Cys99 in TIMP-1. Continuing along this segment, the chain folds helically (α-helix 1) and then proceeds into the proper β-barrel motif at strand A (Fig 2) [14-16,19,23,25]. It has been shown that the residues at the molecular edge critically contribute to defining the specificity and affinity of the inhibitory binding to the MMPs. Mutational analysis of Thr2 in TIMP-1 and Ser2 in TIMP-4 has shown that size, charge and polarity of residue two in the TIMP structure is a major determinant of MMP inhibition [16,23,27,29-32].

From the alignment shown in Fig. 1, it is apparent that in TIMP-4 the β-hairpin connecting the β-strands A and B (AB-loop) differs in sequence and length relative to the corresponding region in the other TIMPs. This loop is one residue shorter than in TIMP-2 and six and five residues larger than in TIMP-1 and TIMP-3, respectively. Crystallographic, NMR and site-directed mutagenesis studies have revealed that the AB-loop is variably involved in the contacts between TIMPs and MMPs [14-16,23]. It has been shown that deleting this loop dramatically decreases the rate of association and increases the equilibrium constant of TIMP-2 binding to the catalytic domain of MT1-MMP (MMP-14) [33]. It was demonstrated that Tyr36, which is unique within the TIMP-2 sequence and located at the tip of the AB-loop, critically contributes to the interactions with MT1-MMP [34]. Notably, the deletion of the AB-loop in TIMP-4 exerts a considerably smaller effect in the kinetics and thermodynamic of binding with MT1-MMP, suggesting a different contribution of the TIMP-4 AB-loop for the MT1-MMP binding [33]. TIMP-4, which associates with the catalytic domain of MT1-MMP at a 20-fold slower rate than TIMP-2, has Ala instead of Tyr at position 36. Moreover, TIMP-4 has two Pro residues at positions relevant for the AB-loop that are not present in the other TIMPs. Pro30 is located at the beginning and Pro35 at the tip of the AB loop. In addition, Pro39 distinguishes the AB-loop of TIMP-2 and TIMP-3 but is substituted for Thr in TIMP-4 (Fig 1). These characteristics of the TIMP-4 AB-loop sequence, that presumably induce local differences respecting to TIMP-2 AB-loop, could also be of relevance for MT1-MMP binding. Remarkably, it was shown that grafting the entire TIMP-2 AB-loop to TIMP-4 transfers the MT1-MMP binding functionality of TIMP-2 AB-loop to TIMP-4. Improvement in the MT1-MMP-binding capacity was also observed when the three residues located at the tip of the TIMP-2 AB-loop, including Tyr36, were transferred to TIMP-4 (Fig. 1). It is of note that in both cases, Pro30 and Pro35 in TIMP-4 were replaced by the corresponding residues in TIMP-2 [33].

It is known that TIMP-4 binds to pro-MMP-2 as TIMP-2 does and is also a strong inhibitor of MT1-MMP; however, notwithstanding the sequence similarity between both inhibitors, TIMP-4 is unable to promote the activation of the pro-MMP-2 by free MT1-MMP enzyme [35]. This action is uniquely facilitated by TIMP-2 and is proposed to require the formation of a membrane ternary complex that includes TIMP-2, pro-MMP-2 and MT1-MMP [36-38]. The crystallographic study of the complex formed by TIMP-2 and pro-MMP-2 has shown that it involves the interaction of the C-terminal domain of the inhibitor with the hemopexin-like domain of the zymogen [17]. Sequence differences at the C-terminal domain between TIMP-4 and TIMP-2 have been suggested to be the cause of the inability of TIMP-4 to promote the activation of pro-MMP-2. TIMP-4 lacks the Met149, Glu192 and Asp193 residues that have been proposed to be key in stabilizing the interactions of the C-terminal domain of TIMP-2 with the hemopexin-like domain of pro-MMP-2. In TIMP-4, Glu192 and Asp193 are replaced by Val and Gln, respectively. In addition, Met149, around which the main cluster of hydrophobic interactions between TIMP-2 and pro-MMP-2 focuses, is replaced in TIMP-4 by Thr. In support of this suggestion, it was recently demonstrated that, in clear contrast with TIMP-2, TIMP-4 is unable to form *in vitro *a stable complex with pro-MMP-2 in presence of the active form of MT1-MMP [33].

At present, six non-human TIMP-4 sequences, five of them from mammals, have been reported [39-42] (Fig 1). Those of mammalian origin are 90 to 100% identical to hTIMP-4, with the chimpanzee TIMP-4 (paTIMP-4) the most closely related. In contrast, the TIMP-4 of the fish Takifugu rubripes (tkTIMP-4) shows only 55% identity to hTIMP-4 [42], a value that compares with the similarity between hTIMP-2 and hTIMP-4. Interestingly, tkTIMP-4 and hTIMP-2 are 50% identical at the sequence level, which is in agreement with the proposed hypothesis of a relatively close common ancestor for both TIMP-2 and TIMP-4 (Fig 1 and Table 1) [8,42,43].

### Regulation

Although the four members of the TIMP family are very similar in structure, they present marked differences in their expression pattern. While TIMP-2 expression is constitutive and ubiquitous, TIMP-1, -3 and -4 expression is inducible and tissue specific. TIMP-1 is mainly expressed in the reproductive system; TIMP-3 in the heart, kidney, and thymus, and TIMP-4 in heart, kidney, pancreas, colon, testes, brain and adipose tissue [7]. TIMP genes have been found in species ranging from *Caenorhabditis elegans *to humans, suggesting a important and conserved role in metazoans [8]. Interestingly, TIMP-1, 3 and 4 genes are nested within introns of the synapsin gene family [44]. Since synapsins are neuronal-specific phosphoproteins involved in synaptogenesis and neurotransmitter release, it is possible that TIMPs could have a regulatory role in the brain. Characterization of mouse TIMP-4 promoter showed potential sites for myogenin, GATA and Ets family members. Interestingly, it lacks AP1 or AP2 sites, which are involved in the inducible and basal expression of TIMP-1, -2 and -3 genes [45]. Additionally, the TIMP-4 promoter contains an estrogen response element (ERE), in accordance with the observed regulation by estrogen during endometrial normal cycle and preimplantation period [46,47]. Similar to TIMP-3, TIMP-4 expression is regulated by promoter methylation in cancer cell lines and tumor samples [48]. In addition, a polymorphism in the region that transcribes the 3' UTR of the TIMP-4 has been identified. The effect of this polymorphism on TIMP-4 expression and function is unknown, however it is associated with a major risk for osteoarthritis in the Korean population [49]. TIMP-4 function is also regulated by postranslational modifications. Peroxinitrite-induced nitration and oligomerization impairs the inhibitory effects of this protein on MMP-2. In this case, four tyrosine residues on TIMP-4 are modified by peroxynitrite exposure. Since peroxynitrite, via post-translational modifications of target proteins, contributes to cardiovascular injury and cancer, its effect on TIMP-4 could be involved in the pathology of these diseases [50].

### Cellular functions

The most widely studied function of TIMPs is their inhibition properties on MMPs. Members of the MMP family include the classical MMPs, the membrane-bound MMPs (MT-MMPs), the ADAMs (a disintegrin and metalloproteinase) and the ADAMTS (a disintegrin and metalloproteinase with thrombospodin motif) [51]. These enzymes and their specific inhibitors are involved in tumor progression and metastasis by regulating extracellular matrix degradation [4]. As mentioned above, TIMP-4 is more closely related to TIMP-2 and -3, than to TIMP-1. Accordingly, the catalytic domain of MMP-19 can be inhibited by TIMP-2, -3 and -4, while TIMP-1 is less efficient [52] (Table 2). Additionally, MMP-26 is inhibited by the same group of TIMPs, and the co-expression of these proteins has been observed in endometrial carcinoma [53] and in breast ductal carcinoma in situ (DCIS) [54]. Enzymatic kinetics studies revealed IC50 values of 19, 3, 45, 8, 83, and 0.4 nM for MMP-1, MMP-2, MMP-3, MMP-7, MMP-9 and MMP-26, respectively, thus demonstrating that TIMP-4 has highest affinity for MMP-26 among these MMPs [55-57] (Table 2). A special case is TIMP-2, which is the only member of the family able to both inhibit and activate MMPs. Although TIMP-4 is able to form a complex with MT1-MMP and the hemopexin domain of MMP-2, it cannot activate the metalloproteinase. However, TIMP-4 can regulate the activity of MMP-2 by efficient inhibition of MT1-MMP-mediated activation and by inhibiting the activated enzyme [35,58,59].

**Table 2:** Inhibitory activity of TIMP-4 on matrix metalloproteinases.

TIMP-4	Target MMP	Kinetic parameter	Key Residue(s)	Ref.
full-length	cdMMP-1^1^	*K*_*i *_2.59 nM	?	[57]
full-lenght	cdMMP-1^1^	IC_50 _19 nM	?	[55]
full-lenght	MMP-1	*K*_*i *_0.65 nM	?	[57]
N-TIMP-4^2^	MMP-2	*K*_*i*_^app ^0.3 nM	?	[33]
full-lenght	MMP-2	*K*_*i*_^app ^≤ 9 pM	?	[35]
full-lenght	MMP-2	IC_50 _3 nM	?	[55]
full-lenght	cdMMP-3^1^	*K*_*i *_1.24 nM	?	[57]
full-lenght	MMP-3	IC_50 _45 nM	?	[55]
full-lenght	MMP-3	*K*_*i *_1.51 nM	?	[57]
full-lenght	MMP-7	IC_50 _8 nM	?	[55]
N-TIMP-4	cdMMP-8^1^	*K*_*i *_9.7 nM	Ser2	[32]
N-TIMP-4	cdMMP-9^1^	*K*_*i *_14.5 nM	Ser2	[32]
full-lenght	MMP-9	IC_50 _83 nM	?	[55]
full-lenght	MMP-26	IC_50 _0.4 nM	?	[56]
N-TIMP-4	mbMT1-MMP^3^	*K*_*i*_^app ^1.0 nM	?	[33]
full-lenght	sMT1-MMP^3^	*K*_*i*_^app ^0.1 nM	?	[35]
N-TIMP-4	ADAM-17/TACE	*Ki *8.68 nM	?	[65]
full-lenght	ADAM-17/TACE	*Ki *180 nM	?	[65]
full-lenght	cdADAM33^1^	*Ki *220 nM	?	[62]

The table shows the kinetic parameter of the TIMP-4 target matrix metalloproteinase (MMP) and, when available, the key residue involved in the interaction.

^1 ^Catalytic domain of the metalloproteinase.

^2 ^Amino-terminal domain of TIMP-4.

^3 ^mbMT1-MMP and sMT1-MMP refer to membrane-bond and soluble forms of Membrane Type 1 Matrix Metalloproteinase, respectively.

*K*_*i *_= Inhibitory constant; IC_50 _= Median inhibitory constant; *K*_*i*_^app ^= Apparent inhibitory constant. ? = not studied.

TIMPs can also inhibit members of the ADAM and ADAMTS families. ADAMTSs are structurally and evolutionarily more related to the ADAM family, and more distantly to MMPs [60]. TIMP-4 and -3 can inhibit the proteolytic activity of ADAM28 on insulin-like growth factor binding protein-3 (IGFBP-3) [61]. Protease activity of ADAM33 is also inhibited moderately by TIMP-3 and -4 and weakly inhibited by TIMP-2 but not by TIMP-1 [62]. In contrast, proteolytic activity of ADAM10 on myelin basic protein can be inhibited by TIMP-1 and -3, while TIMP-2 and -4 were unable to inhibit this activity [63]. TIMP-3 is also a good inhibitor of ADAM17, while TIMP-2 and -4 are weak inhibitors, TIMP-1 did not show inhibition [64]. However a TIMP-4 mutant, in which three residues in the AB loop were replaced by surface residues of TIMP-3, showed a ten-fold increase in the binding affinity to ADAM17 [65]. For ADAMTS4, TIMP-3 is the most efficient inhibitor, followed by TIMP-1 and -2, while TIMP-4 is a less efficient inhibitor [66]. ADAMTS2 is only inhibited by TIMP-3, while TIMP-1, -2 and -4 did not show inhibitory activity [67]. Although ADAMTS5 is inhibited by TIMP-3, TIMP-4 is a weak inhibitor and no inhibition by TIMP-1 and -2 was observed [68]. Platelets contain and release several members of the TIMP, MMP and ADAM families, including MMP-1, MMP-2, MMP-3, MMP-9, MT1-MMP (MMP-14), ADAM10, ADAM17, ADAMTS13, TIMP-1, TIMP-2 and TIMP-4. These proteins regulate platelet functions such as agonist-stimulated platelet adhesion and aggregation, tumor cell-induced platelet aggregation and platelet-leukocyte aggregation [69]. TIMP-4 has been identified as the major MMP inhibitor in human platelets [70], so it is possible that this protein has an important regulatory role in these phenomena.

In addition to their inhibitory actions, TIMPs have independent cell signaling functions that modulate angiogenesis, proliferation and apoptosis [71]. Mutants that lack the ability to inhibit MMPs but retain the capacity to modulate specific cellular functions [72] have substantiated the MMP-independent activities of both TIMP-1 and TIMP-2. It has been shown that one of these mutants, Thr2Gly-TIMP1, is able to protect MCF10A human breast epithelial cell from apoptosis as efficiently as the wild type TIMP-1 [71]. Similarly, a TIMP-2 mutant containing an appending Ala residue at the amino terminus (Ala+TIMP-2) binds the surface of human A549 cancer cells with high affinity and retains an *in vitro *cell growth-inhibitory activity similar to the TIMP-2 wild type protein regardless of being a non-active MMP-inhibitor [73]. Recently, a mayor breakthrough in MMP-independent activities was achieved by the discovery of binding partners for TIMP-1, 2 and 3 as CD63, integrin a3b1 and VEGF receptor-2, respectively, and putatively CD63 for TIMP-4 [74]. Although not firmly established, the TIMP-4 association could imply a similar role to that of TIMP-1, that is, the ability to activate integrin b1 complex and promote survival signaling pathways such as FAK, Src, PI 3-K and MAPK. TIMP-4 mutants that lack MMP-inhibitory activity could be helpful in elucidating the MMP-dependence or independence of normal and cancer cells' behavior [72]. This goal would be greatly facilitated by studies around the structural basis of TIMP-4 MMP-inhibitory specificity. TIMP-4 has proven to be difficult to fold efficiently *in vitro *from bacterial inclusion bodies but it can be expressed in mammalian cells [35], baculovirus [55], and yeast [32], in a quantity that could facilitate the production of crystals suitable for structural studies. For obvious reasons, determining the 3-D structure of free TIMP-4 as well as in complex with relevant MMPs will be an important forward step. Site-directed mutagenesis studies as well as the construction of chimeras by domain-exchange could be helpful in identifying which region at the N- and/or C- terminal domains of TIMP-4 are involved in binding to the putative cellular receptor.

### Role in cancer progression

Tumor microenvironment is key to cancer progression. To survive and to achieve their full malignant phenotype, cancer cells require signaling via adhesion molecules with surrounding non-neoplastic cells, paracrine loops of released signaling factors and extracellular matrix interaction and degradation.

In particular, the extracellular matrix (ECM) presents not only as a structural impediment to neoplastic cells' migration, but also contributes to their behavior by providing biochemical clues such as growth factor and cytokines that are anchored to it. Matrix metalloproteinases (MMPs), a disintegrin and metalloproteinases (ADAMs) and tissue inhibitors of metalloproteinases (TIMPs) constitute the major proteolytic axis of ECM, and thus, are critical for cancer progression.

For these reasons, it is not unexpected that TIMP-4 is disregulated during cancer progression of several organs. Elevated levels of TIMP-4 have been found in breast, ovary, cervical, prostate, brain, colon, endometrium and papillary renal tumors, whereas down-regulation was observed in pancreatic and clear cell renal tumors (Table 3) [75-78]. This list is not comprehensive, since an expression profile in the Oncomine database [79], which contains microarray expression data for multiple cancer types, shows that other cancer types also present up-regulated levels of TIMP-4, including oligodendrogliomas and astrocytomas, seminomas and hairy cell leukemias (Table 4).

**Table 3:** TIMP-4 expression in human cancer.

**Cancer**	**Assay**	**Expression**	**Reference**
Breast	IHC	↑ in early stages, ↓ in advanced stages	[54]
Ovary	IHC	↑	[78]
Cervical	RT-PCR	↑	[77]
Prostate	IHC	↑ in early stages, ↓ in advanced stages	[83]
Gliomas	RT-PCR	↑ in early stages, ↓ in advanced stages	[76]
Pancreas	IHC	↓	[75]
Colon	IHC	↑	[80]
Endometrium	IHC	↑	[46,53]
Kidney Papillary	RT-PCR	↑	[81]
Kidney Clear cell	RT-PCR	↓	[81]

Published research.

Up and down arrows represent increased and decreased expression, respectively

**Table 4:** TIMP-4 expression in human cancer.

**Cancer**	**Differential expression**	**# Studies**	**P-value**
Glioblastoma multiforme	↑	2	1.2E-5 to 1.8E-17
Oligodendroglioma	↑	2	1.5E-11 to 7.8E-17
Astrocytoma	↑	2	5.3E-5 to 1.4E-16
Hairy cell leukemia	↑	1	7-1E-8
Seminoma	↑	1	3.1E-13
Bladder	↓	2	2.2E-5 to 9.8E-8
Head and Neck	↓	1	2.4E-7
Prostate	↓	1	3.1E-7

Oncomine database.

Up and down arrows represent increased and decreased expression, respectively. P-value threshold was set to 1E-4

Interestingly, down-regulated levels were found in bladder, prostate and head and neck cancer. Three possible reasons, not mutually exclusive, could explain these opposing results. First, the methods used may account for some of the results. Since it has been shown that there are different TIMP-4 protein pools [80], mRNA expression analyses may not accurately reflect protein levels at a particular time point. Second, disease or tissue-specific differences may account for some of the results. This is exemplified by renal cell carcinoma, in which TIMP-4 is elevated in papillary cancer cells, in contrast with clear cell carcinoma, in which TIMP-4 mRNA is reduced [81]. Finally, temporal expressional changes during cancer progression could also explain the differences. Thus, analysis of samples at later stages may detect only a TIMP-4 decrease, missing early changes, especially when the studies lack samples from initial cancer stages or normal tissue controls. Supporting this scenario, it has been shown that TIMP-4 increases in initial stages of prostate, breast and glial tumors, decreasing at later stages (Table 3). A decrease in expression at later stages has also been found for pancreatic and endometrial tumors.

Clearly, additional studies using a wider range and number of samples are needed to draw a conclusion, but, for some tumors, a scenario in which TIMP-4 marks the transition to invasive cancer emerges. This transition seems also to be correlated with the expression of MMP-26, a metalloproteinase inhibited by TIMP-4, providing an important invasive proteolytic axis [54]. Since MMP-26 promotes invasion by dissolving basement membrane after activation of MMP-9 [82], TIMP-4 may be acting as a natural anti-invasive protein by preventing activation of MMP-2 and 9, in a direct and indirect manner, as described previously. This coexpression has been clearly demonstrated in prostate [83], breast [54] and endometrial carcinomas [53], with the last two associated with the estrogen receptor signaling pathway. Nevertheless, there are two conflicting results. First, in contrast to early reports on the antitumoral function of TIMP-4 on breast cancer tumor growth and invasion [84], Jiang et al. reported that systemic delivery of TIMP-4 stimulated breast cancer tumorigenesis [85]. Although conclusive for the particular cell line, this last report is not valid, since there is ample evidence that the cell line used, MDA-435, is really a melanoma line [86]. These results urgently call for a new *in vivo *study. Second, Savinov et al. [87] have shown that MMP-26 up-regulation in breast ductal carcinoma *in situ *correlates with longer patient survival, perhaps due to the intracellular cleavage of ER-beta by this protease. These authors later propose that the MMP-26 mislocalization may be inhibiting cancer progression by its effects on ER-beta [88]. Since TIMP-4 was not measured, no conclusion regarding TIMP-4 correlation with breast cancer progression can be made, although further studies on the possible dual role of this inhibitor are warranted.

All these results point toward the need to perform additional studies to determinate the relevance of TIMP-4 in cancer invasion and progression, in particular to establish if a positive correlation with prognosis or inverse correlation with invasion in patients can be made. In addition, in light of the problems with previous models, the possible use of TIMP-4 as a therapeutic target should not be dismissed. Finally, it is important to study the potential relevance of the possible new transduction partners of TIMP-4 on cancer progression and to identify the importance of their localization.

## Conclusion

Compared to other members of the family, TIMP-4 has received less attention than deserved, perhaps due to the restricted tissue expression and suspected pro-tumoral activities. Nevertheless, the recent reports showing a correlation between its expression and invasion and the problems with the *in vivo *models warrant additional efforts to establish the relevance of this protein in cancer. In particular, additional studies are needed to elucidate its molecular functions and intracellular role(s), analyze its potential as a biomarker and to test if it could be a therapeutic target. These efforts surely will get TIMP-4 the attention it needs from the scientific community.

## Competing interests

The authors declare that they have no competing interests.

## Authors' contributions

JM-Z coordinated the work, wrote several parts of the article and proofreaded the manuscript, LDP wrote the structure section, GC wrote the regulation section and VM wrote the cancer progression section. All authors read and approved the final manuscript.
